# *TGFBR1* variants TGFBR1^*^6A and Int7G24A are not associated with an increased familial colorectal cancer risk

**DOI:** 10.1038/sj.bjc.6605054

**Published:** 2009-04-28

**Authors:** J Skoglund Lundin, J Vandrovcova, B Song, X Zhou, M Zelada-Hedman, B Werelius, R S Houlston, A Lindblom

**Affiliations:** 1 Department of Molecular Medicine and Surgery, Karolinska Institutet, CMM L8:02, S-171 76 Stockholm, Sweden; 2 Department of Pathology, Dalian Medical University, Dalian 116027, China; 3 Section of Cancer Genetics, Institute of Cancer Research, SM2 5NG, Sutton, Surrey, UK

**Keywords:** colorectal cancer, adenoma, TGFBR1, TGFBR1^*^6A, Int7G24A

## Abstract

Variants of the transforming growth factor-beta receptor type 1 (*TGFBR1*) gene, TGFBR1*6A and Int7G24A, have been suggested to act as low-penetrance tumour susceptibility alleles with TGFBR1*6A being causally responsible for some cases of familial colorectal cancer (CRC). We performed a case–control study of 262 unrelated familial CRC cases; 83 hereditary non-polyposis colorectal cancer (HNPCC) and 179 non-HNPCC. Patients were genotyped for TGFBR1*6A and Int7G24A and compared with 856 controls. Further, we screened the coding region of *TGFBR1* in affected members of a large family with CRC linked to 9q22.32-31.1. TGFBR1*6A allelic frequency was not significantly different in all of the familial cases compared with controls (0.107 and 0.106, respectively; *P*=0.915). In a subgroup analysis allele frequencies were, however, different between HNPCC and non-HNPCC familial cases (0.157 and 0.084, respectively; *P*=0.013). TGFBR1*6A genotype did not influence age of onset. Int7G24A allele frequencies were similar in cases and controls. No germ-line mutation was identified in the family with CRC linked to this chromosomal region. Our study provides no substantial support for the hypothesis that the polymorphic variants TGFBR1*6A or Int7G24A contribute to familial CRC risk. We cannot, however, exclude the possibility that *TGFBR1* variants have a modifying effect on inherited risk *per se*.

Colorectal cancer (CRC) is the third most common cause of cancer-related mortality in the Western countries, and in the United States it represents the second most common cause of cancer mortality (Parkin, 2001). Although 35% of all CRC seems to involve an inherited susceptibility (Lichtenstein *et al*, 2000). Mendelian predisposition syndromes associated with mutations in known genes (such as *APC*, DNA mismatch repair genes (MMR), *MYH*, *SMAD4*, *BMPR1A*/*ALK3* and *STK11*/*LKB1*) collectively only account for 5% of disease burden (Lichtenstein *et al*, 2000; Lynch and de la Chapelle, 2003). The nature of the remaining heritability is undefined, but a model in which part of the inherited risk is conferred by common, low-penetrance alleles seems highly probable and has been the rationale for seeking to identify disease alleles through association analyses.

Transforming growth factor-*β* (TGF-*β*) is a potent inhibitor of cell growth influencing the behaviour of a number of cancers (Derynck *et al*, 2001). The TGF-*β* mediates its action through a heteromeric cell-surface complex of two types of transmembrane serine/threonine kinases, TGF-*β* receptor type 1 (TGFBR1) and type 2 (TGFBR2) (Lin *et al*, 1992; Ebner *et al*, 1993). The *TGFBR2* gene is mutated in several cancer types with ∼90% of colon cancers characterized by MMR deficiency displaying frameshift mutations in a polyadenine tract of TGFBR2, which encodes the signal peptide of the expressed protein (Parsons *et al*, 1995).

A relatively common variant of the *TGFBR1* gene, TGFBR1*6A, is caused by deletion of three GCG triplets coding for alanine within a nine alanine (^*^9A) repeat sequence of exon 1, reportedly associated with less TGF-*β* growth inhibitory signalling capacity than the native expressed protein (Chen *et al*, 1999; Pasche *et al*, 1999). In some epidemiological studies, the TGFBR1*6A allele has been reported to be associated with an increased risk of a number of different malignancies, including CRC (Pasche *et al*, 1999; Chen *et al*, 2001; Baxter *et al*, 2002; Kaklamani and Pasche, 2005). In addition, the TGFBR1*6A variant has been proposed to be directly causally responsible for a proportion of hereditary non-polyposis colorectal cancer (HNPCC), especially those without MMR deficiency (Bian *et al*, 2005). Intriguingly, *TGFBR1* maps to chromosome 9q22.32-31.1, a from several independent studies suggested CRC susceptibility locus (Wiesner *et al*, 2003; Kemp *et al*, 2006; Skoglund *et al*, 2006).

Another polymorphic variant of *TGFBR1*, Int7G24A, has also been implicated in cancer susceptibility, associations with kidney, bladder, breast and non-small cell lung cancer being reported (Chen *et al*, 1999, 2004; Zhang *et al*, 2003).

To further evaluate the relationship between the *TGFBR1* variants and CRC risk we determined whether these variants contribute to familial CRC. Using a case–control design, we compared TGFBR1*6A and Int7G24A allele frequencies in HNPCC and non-HNPCC familial CRC cases with population-based controls. We also examined whether germ-line *TGFBR1* mutations are responsible for the CRC susceptibility locus on chromosome 9 by screening the entire coding region of *TGFBR1* in affected members of a large family with adenoma and CRC linked to chromosome 9q22.32-31.1 (Skoglund *et al*, 2006).

## Materials and methods

### Study population

Two hundred and sixty-two families, ascertained through the Family Cancer Clinic at Karolinska University Hospital, Stockholm, Sweden during 1990–2006, were included in the study. A total of 83 families, all with a germ-line mutation in one of the MMR genes, were considered as HNPCC and 179 families were diagnosed and counselled as non-HNPCC according to our earlier published protocol (Lagerstedt Robinson *et al*, 2007). From each family one case was selected for case–control studies. The selection was made using the following priority order; (1) proband (when affected), (2) youngest CRC case or (3) youngest case with adenoma. Statistical analysis was carried out on CRC plus adenoma cases and CRC cases alone. Epidemiological studies have shown that a personal history of colon adenomas places one at increased risk of developing CRC (Neugut *et al*, 1993; Jacobson and Neugut, 1996; Liljegren *et al*, 2003). Furthermore, adenomas are over-represented in CRC families and first-degree relatives of patients with large adenomas are at increased risk of developing CRCs or large adenomas (Lindgren *et al*, 2002; Cottet *et al*, 2007). Removal of adenomatous polyps is associated with reduced CRC incidence (Muller and Sonnenberg, 1995; Winawer *et al*, 2000). Eight hundred and fifty-six blood donors from the Karolinska University Hospital, Stockholm, Sweden served as source of control DNA.

For mutation screening of *TGFBR1*, we analysed genomic DNA from a family with CRC and adenoma linked to chromosome 9q22.32-31.1. A full description of this family has been published earlier (Skoglund *et al*, 2006).

The study was undertaken in accordance with the Swedish legislation of ethical permission (2003:460) and the Stockholm regional ethical committee (Dnr: 2000/291, 2005/566) in accordance with the Declaration of Helsinki.

### Genotyping

**Figure 1:**
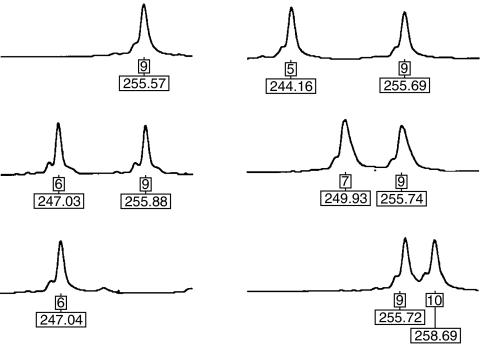
Representative electropherograms of the ^*^9A/^*^9A, ^*^6A/^*^9A, ^*^6A/^*^6A, ^*^5A/^*^9A, ^*^7A/^*^9A and ^*^9A/^*^10A genotypes. Allele and size in base pairs are indicated below each peak.

The TGFBR1*6A variant was determined by PCR amplification using fluorescently labelled primers Fwd- 5′-GAGGCGAGGTTTGCTGGGGTGAGG-3′ and Rev- 5′-CATGTTTGAGAAAGAGCAGGAGCG-3′. Amplification was performed using the PlatinumTaq DNA polymerase and supplied protocol for GC-rich fragments (Invitrogen, Carlsbad, CA, USA). Amplified fragments were separated by electrophoresis on an ABI 377 semi-automated DNA Sequencer (Applied Biosystems, Bedford, MA, USA) and genotypes assigned using GENESCAN and GENOTYPER software (Applied Biosystems). A product size of 256 bp corresponded to the most common allele, ^*^9A, whereas a product size of 247 bp corresponded to the ^*^6A allele. Besides the ^*^9A and ^*^6A alleles, we observed three rare alleles (Figure 1). The ^*^5A/^*^9A genotype was detected in one non-HNPCC case and one control case. Another two individuals with a ^*^7A/^*^9A genotype and one individual with a ^*^9A/^*^10A genotype was detected, all in the control group. All these rare alleles have been reported earlier (Pasche *et al*, 1999; Spillman *et al*, 2005). All ^*^6A homozygotes and ^*^6A/^*^9A heterozygotes and all samples with rare alleles were retyped after a second independent PCR amplification to confirm the allele calling.

**Figure 2:**
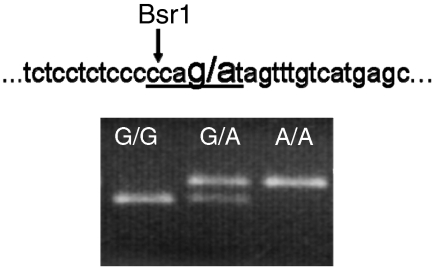
Genomic sequence surrounding the Int7G24A variant and a representative photograph of the PCR-RFLP analysis. The variant site is marked in bold and the Bsr1 recognition site is underlined. Bsr1 recognises and cuts the wild-type sequence (smaller band) whereas the variant abolishes the Bsr1 restriction site (larger band).

Genotyping of the Int7G24A variant was performed by PCR amplification of intron 7 using primers Fwd- 5′-GGAGGTTCATCCAAATATGGC-3′ and Rev- 5′-CTCTGGCACTCGGTGACAT-3′ followed by Bsr1 digestion and visualisation on a 2.5% agarose gel (Figure 2). For 85 samples, restriction enzyme digestion was replicated after a second independent PCR amplification to confirm the allele calling. Further details on genotyping can be obtained from the authors.

### Mutation screening of *TGFBR1*

Mutational analysis of *TGFBR1* was conducted on germ-line DNA from affected individuals in family 24 carrying the linked haplotype using a combination of denaturing high performance liquid chromatography (dHPLC) (exons 2–9+1091 bp upstream of exon 1) and direct sequencing (exon 1+3′UTR). Details on family 24 have been published earlier (Skoglund *et al*, 2006). Owing to stringent surveillance only one individual alive (Co-166) had developed CRC. Individual Co-166 was mutation screened for all fragments. The selection of additional individuals for mutation screening was carried out based on carrier status of the linked haplotype along with grade of affected status.

Screening for genomic deletions and rearrangements was performed using reverse transcriptase–PCR. RNA from two affected individuals (Co-186 and Co-213) plus two controls was extracted with the Qiagen RNeasy kit (Operon Biotechnologies, Huntsville, AL, USA) and reverse-transcribed with a first-strand synthesis kit (Amersham Biosciences, Piscataway, NJ, USA). Primer sequences and details of all the assays are available on request.

### Statistical methods

Risks associated with *TGFBR1* genotypes were estimated by odds ratios (ORs) using unconditional logistic regression, and associated 95% confidence intervals (CIs) were computed. To test for population stratification the distribution of genotypes were tested for a departure from Hardy–Weinberg equilibrium. Differences in the distribution of continuous variables were compared using Mann–Whitney *U*-test or the statistics of Cuzick (1985) and Altman (1991) and between proportions by Fisher's exact test. Confidence limits for sequence changes among cases were computed under the assumption that frequencies followed a Poisson distribution. All statistical analyses were performed using STATA Version 7.0 (Stata Corporation, College Station, TX, USA). A *P*-value of 0.05 was considered statistically significant in all analyses.

## Results

The characteristics of the study participants are detailed in Table 1. Cases from HNPCC families were diagnosed with CRC younger than those from non-HNPCC families (45 years *vs* 57 years, respectively; *P*<0.01) consistent with ascertainment selection.

The observed frequencies of TGFBR1*6A genotypes in cases and controls were in accordance with Hardy–Weinberg laws of equilibrium (*P*=0.35 and 1.00 in familial non-HNPCC and HNPCC cases; *P*=0.86 in controls), providing no evidence of population stratification within the dataset. The frequency of TGFBR1*6A was not significantly different between controls (0.106, 95% CI: 0.102–0.135) and all familial cases (0.107, 95% CI: 0.082–0.137; Table 2A). Confining the affection status to a diagnosis of CRC only and excluding adenomas did not change the results much (0.117, 95% CI: 0.087–0.151; Table 2B).

Eighty-three of the cases had HNPCC and 179 had non-HNPCC hereditary CRC. Among those with non-HNPCC; 26 (14.5%) and two (1.1%) were hetero- and homozygous for the TGFBR1*6A allele, respectively (TGFBR1*6A allele frequency 0.084). Of the 83 cases with HNPCC; 22 (26.5%) and two (2.4%) were TGFBR1*6A heterozygotes and homozygotes, respectively (TGFBR1*6A allele frequency 0.157). Of the 24 TGFBR1*6A carriers; 10 had a *MLH1* mutation, 11 had a *MSH2* mutation and three had a *MSH6* mutation. Although the frequency of the TGFBR1*6A allele was similar in non-HNPCC familial cases and controls (0.084 and 0.106, respectively; *P*=0.23), the frequency in HNPCC cases was markedly elevated (0.157; *P*=0.045) compared with the controls. Hence, there was an apparent difference in TGFBR1*6A allele frequency between HNPCC and non-HNPCC familial cases (0.157 and 0.084, respectively; *P*=0.013). Table 2A details the ORs of CRC and adenoma and corresponding 95% CIs associated with TGFBR1*6A hetero- and homozygosity in the two familial CRC groups. When performing test for association restricting affection status in cases to CRC (non-HNPCC, *n*=132; HNPCC, *n*=82), corresponding ORs were similar (Table 2B).

To further explore the possibility that carrier status might affect CRC risk we compared the age of onset of CRC in TGFBR1*6A carriers and non-carriers. There was no association between age at diagnosis of CRC and TGFBR1*6A genotype (data not shown). Comparison of the cumulative distribution curves also showed no significant difference in carriers compared with non-carriers. Among familial non-HNPCC cases the average age at cancer diagnosis in TGFBR1*6A carriers and non-carriers was 58.4 years (s.d., 13.0) and 56.8 years (s.d., 10.6), respectively. Corresponding ages at diagnosis in carriers and non-carriers in HNPCC cases was 43.3 years (s.d., 11.0) and 45.7 years (s.d., 10.6), respectively.

In the study by Bian *et al* (2005), the highest TGFBR1*6A frequency was found among MMR mutation-negative cases with MSI-negative tumours. Data on tumour DNA samples evaluated for MSI status were available from 249 of the cases. Among all familial cases, TGFBR1*6A carrier frequency was not significantly different in MSI-positive cases compared with MSI negative (*P*=0.17). Among HNPCC cases with MSI tumours (MMR/MSI positive) over-representation of TGFBR1*6A carriers was evident, albeit non-significantly compared with the controls (data not shown). Subdividing the whole sample set by Amsterdam criteria and MMR status showed the highest TGFBR1*6A allelic frequency in Amsterdam criteria-positive families with a detected MMR gene mutation (0.198). On the contrary, the allelic frequency was zero in Amsterdam criteria-negative families with no detected MMR mutation.

The Int7G24A variant was successfully genotyped in 262 familial CRC cases, 179 non-HNPCC and 83 HNPCC, and 853 controls. The observed frequencies of Int7G24A genotypes in cases and controls were in accordance with Hardy–Weinberg laws of equilibrium (*P*=1.00 and 0.09 in familial non-HNPCC and HNPCC cases; *P*=0.82 in controls). There were no differences in allele or genotype frequencies between cases and controls or between the different types of familial CRC (Tables 3A and 3B).

In an earlier study, we have shown linkage of CRC and colorectal adenomas to chromosome 9q22.32-31.1 in a large Swedish family (family 24) (Skoglund *et al*, 2006). To exclude the possibility that sequence variation in *TGFBR1* is responsible for the linkage in the family, we screened for germ-line mutations in affected family members. The TGFBR1*6A and Int7G24A variants were not on the linked haplotype. Two individuals (Co-213 and Co-219) were heterozyogous TGFBR1*6A carriers. The remaining seven individuals with the linked haplotype were ^*^9A homozygous. For the Int7G24A variant; the two individuals screened for this fragment (Co-166 and Co-648) were not carriers of this variant. No sequence change was detected on the linked haplotype.

## Discussion

Some studies have reported an over-representation of TGFBR1*6A in individuals with a number of different cancers fuelling speculation by Pasche *et al* (2004) that the TGFBR1*6A allele represents a low-penetrance allele with pleiotropic effects. The same research group reported a case–control study comparing TGFBR1*6A allelic frequency among HNPCC patients subdivided by first MMR gene mutations status and second tumour MSI status, observing a markedly elevated frequency of TGFBR1*6A carriers among MMR gene mutation-negative cases with the highest frequency among cases with MSI-negative tumours (Bian *et al*, 2005). On this basis they postulated that the TGFBR1*6A allele may be causally responsible for a proportion of non-HNPCC occurrence.

In contrast to Bian *et al* (2005) in our study TGFBR1*6A allelic frequency was not significantly different in all of the familial cases compared with controls. Moreover, in a subgroup analysis (which inevitably invokes the issue of multiple testing and apparent paradoxes) allele frequencies were only significantly different in HNPCC cases with a known MMR genetic defect, indicating a possible role if any as a modifying factor in HNPCC families. Further subdivision by MMR- and MSI status confirms this observation however, groups are small and differences were not significant. Further studies are needed to confirm this observation.

In our study, 48 cases affected with adenomas were included in the analysis. However, as shown in Table 2b excluding these did not change the results indicating that the TGFBR1*6A variant confers the same increased risk for adenoma as for CRC in these families.

The intronic variant, Int7G24A, has been variously associated with an increased risk of kidney-, bladder- and breast cancer (Chen *et al*, 2004, 2006). Although our analyses were based on a smaller control dataset than that used for evaluation of TGFBR1*6A, we found no real evidence that Int7G24A influences CRC risk.

The pivotal role *TGFBR1* plays in tumour development makes the hypothesis that germ-line variation in the gene may influence CRC susceptibility an attractive concept. A recent study has reported that germ-line allele-specific expression (ASE) of *TGFBR1* is a quantitative trait detectable in 10–20% of CRC patients and 1–3% of the population (Valle *et al*, 2008). Moreover it was proposed ASE is dominantly inherited trait, if confirmed these findings are compatible for this association being responsible for >50% of the excess familial risk of CRC. Paradoxically, although two major *TGFBR1* haplotypes were predominant among ASE cases (one of the two major haplotypes included TGFBR1*6A and overall 50% of ASE cases carried the TGFBR1*6A variant) no causal variant was identified.

In contrast to the findings of Valle *et al* (2008) in a large case–control study and meta-analysis of eight earlier published studies of the relationship between TGFBR1*6A and CRC, we found little evidence for the tenet that germ-line variation in the gene defined by this variant significantly influences CRC risk (Skoglund *et al*, 2007).

For this study, we had 262 families available for analysis. On the basis of published estimations of sample size needed for achieving adequate power to detect association our data set has limited power. However, analysing familial cases provide a means of generating a genetically enriched dataset with increased power to show associations compared with using unselected cases (Houlston and Peto, 2003). Without appropriate adjustment, however, derived genotypic risks computed are inflated, hence the upper confidence limits of the risk of CRC associated with the variants in our study will be overoptimistic.

As source of controls, we have employed blood donors. No data, because of policy of anonymity, was available for age, ethnicity, medical- or family history for these individuals. However, it is unlikely that this will have significantly influenced study findings as any age or gender difference from controls will be minimal. Furthermore, although the ethnicity data on blood donors was not available these individuals were drawn from the same demographic region as the cases analysed making the probability of confounding from population stratification unlikely. Blood donors constitute a healthy cohort with a risk for later onset diseases, such as CRC close to that of the general population. Still, even if the controls are representative for the general population, cases included are not consecutive but constitute a cohort genetically enriched for risk alleles. Therefore, we used OR rather than the relative risk to calculate the risk associated with the variants.

Mutational analysis of *TGFBR1* in the earlier published family 24 with CRC and adenoma linked to chromosome 9q22.32-31.1 (Skoglund *et al*, 2006) showed no sequence changes on the linked haplotype. Furthermore, the linked haplotype carried the normal sequence for the two studied variants, TGFBR1*6A and Int7G24A. Therefore, we can exclude these variants as disease causing in this family. This is in accordance with two recent studies where the TGFBR1*6A variant was investigated in a sample linked to this locus and excluded as the disease-causing variant (Kemp *et al*, 2006; Daley *et al*, 2007). The fact that the linked haplotype carried the normal sequence for the two studied variants TGFBR1*6A and Int7G24A means it is therefore different from at least one of the *TGFBR1* ASE haplotypes reported by Valle *et al* (2008) which carried the TGFBR1*6A variant.

We cannot exclude the possibility that variants of *TGFBR1* are associated with a small CRC risk modifying the impact of other gene effects. However, on the basis of our findings it seems unlikely that variation in *TGFBR1* defined by the TGFBR1^*^6A and the Int7G24A makes a significant contribution to familial CRC risk.

## Figures and Tables

**Table 1:** Demographics and clinical status of HNPCC and non-HNPCC familial colorectal cancer cases

	**HNPCC (*n*=83)**	**Non-HNPCC (*n*=179)**
**Characteristics**	**No. (%) of patients**	**No. (%) of patients**
*Age*		
Mean (s.d.), years	45 (11)	57 (11)
Range, years	22–76	28–84
		
*Sex*		
Male	46 (55)	78 (44)
Female	37 (45)	101 (56)
		
*Clinical manifestation*		
CRC	82 (99)	132 (74)
Adenoma	1 (1)	47 (26)
		
*Fulfillment of Guidelines* a		
Amsterdam criteria I	42 (56)	17 (9)
Amsterdam criteria II	53 (71)	19 (11)
Bethesda Guidelines	73 (97)	140 (78)
		
*MMR gene mutation*		
MLH1	40 (48)	—
MSH2	35 (42)	—
MSH6	8 (10)	—

Abbreviations: CRC=colorectal cancer; HNPCC=hereditary non-polyposis colorectal cancer; MMR=mismatch repair.

a For a definition of the Amsterdam criteria I, see Vasen *et al* (1991); extended Amsterdam II criteria, see Vasen *et al* (1999); Bethesda guidelines, see Rodriguez-Bigas *et al* (1997) and Umar *et al* (2004).

**Table 2a:** Association between TGFBR1^*^6A genotypes and risk of colorectal cancer and adenoma

	**Controls *n*=856^a^**	**All familial cases *n*=262^a^**		**Non-HNPCC *n*=179^a^**		**HNPCC *n*=83**	
**Exon 1Genotype**	**No. of cases (%)**	**No. of cases (%)**	**OR (95% CI)**	**No. of cases (%)**	**OR (95% CI)**	**No. of cases (%)**	**OR (95% CI)**
9A/9A	682 (79.7)	209 (79.8)	1.0	150 (83.8)	1.0	59 (71.1)	1.0
9A/6A	160 (18.7)	48 (18.3)	0.98 (0.68–1.40)	26 (14.5)	0.74 (0.47–1.16)	22 (26.5)	1.59 (0.95–2.67)
6A/6A	10 (1.2)	4 (1.5)	1.31 (0.41–4.21)	2 (1.1)	0.91 (0.20–4.19)	2 (2.4)	2.30 (0.50–10.8)
9A/6A and 6A/6A	170 (19.9)	52 (19.8)	1.00 (0.71–1.41)	28 (15.6)	0.75 (0.48–1.16)	24 (28.9)	1.63 (0.99–2.70)
6A frequency	0.106	0.107		0.084		0.157	

Abbreviation: HNPCC=hereditary non-polyposis colorectal cancer.

a Included in the total number are rare *TGFBR1* alleles such as ^*^5A, ^*^7A, ^*^8A, ^*^10A, ^*^11A and ^*^12A.

**Table 2b:** Association between TGFBR1*6A genotypes and risk of colorectal cancer

	**Controls *n*=856^a^**	**All familial cases *n*=214^a^**		**Non-HNPCC *n*=132^a^**		**HNPCC *n*=82**	
**Exon 1 Genotype**	**No. of cases (%)**	**No. of cases (%)**	**OR (95% CI)**	**No. of cases (%)**	**OR (95% CI)**	**No. of cases (%)**	**OR (95% CI)**
9A/9A	682 (79.7)	167 (78.0)	1.0	109 (83.2)	1.0	58 (70.7)	1.0
9A/6A	160 (18.7)	42 (19.6)	1.07 (0.73–1.57)	20 (15.2)	0.78 (0.47–1.30)	22 (26.8)	1.62 (0.96–2.72)
6A/6A	10 (1.2)	4 (1.9)	1.63 (0.51–5.27)	2 (1.5)	1.25 (0.27–5.79)	2 (2.4)	2.35 (0.50–11.0)
9A/6A and 6A/6A	170 (19.9)	46 (21.3)	1.11 (0.77–1.60)	22 (16.7)	0.81 (0.50–1.32)	24 (29.2)	1.66 (1.00–2.75)
6A frequency	0.106	0.117		0.091		0.159	

Abbreviation: HNPCC=hereditary non-polyposis colorectal cancer.

a Included in the total number are rare *TGFBR1* alleles such as ^*^5A, ^*^7A, ^*^8A, ^*^10A, ^*^11A and ^*^12A.

**Table 3a:** Association between Int7G24A genotypes and risk of colorectal cancer and adenoma

	**Controls *n*=853**	**All familial cases *n*=262**		**Non-HNPCC *n*=179**		**HNPCC *n*=83**	
**Int7G24A Genotype**	**No. of cases (%)**	**No. of cases (%)**	**OR (95% CI)**	**No. of cases (%)**	**OR (95% CI)**	**No. of cases (%)**	**OR (95% CI)**
G/G	559 (65.5)	165 (63.0)	1.0	110 (61.4)	1.0	55 (66.3)	1.0
G/A	265 (31.1)	83 (31.7)	1.06 (0.79–1.44)	61 (34.1)	1.17 (0.83–1.65)	22 (26.5)	0.84 (0.50–1.41)
A/A	29 (3.4)	14 (5.3)	1.64 (0.84–3.17)	8 (4.5)	1.40 (0.62–3.15)	6 (7.2)	2.10 (0.84–5.29)
G/A and A/A	294 (34.5)	97 (37.0)	1.12 (0.84–1.49)	69 (38.5)	1.19 (0.86–1.66)	28 (33.7)	0.97 (0.60–1.56)
A frequency	0.189	0.212		0.215		0.205	

Abbreviation: HNPCC=hereditary non-polyposis colorectal cancer.

**Table 3b:** Association between Int7G24A genotypes and risk of colorectal cancer

	**Controls *n*=853**	**All familial cases *n*=214**		**Non-HNPCC *n*=132**		**HNPCC *n*=82**	
**Int7G24A Genotype**	**No. of cases (%)**	**No. of cases (%)**	**OR (95% CI)**	**No. of cases (%)**	**OR (95% CI)**	**No. of cases (%)**	**OR (95% CI)**
G/G	559 (65.5)	135 (63.1)	1.0	80 (60.6)	1.0	55 (67.1)	1.0
G/A	265 (31.1)	67 (31.3)	1.05 (0.76–1.45)	46 (34.8)	1.21 (0.82–1.79)	21 (25.6)	0.81 (0.48–1.36)
A/A	29 (3.4)	12 (5.6)	1.71 (0.85–3.45)	6 (4.5)	1.45 (0.58–3.59)	6 (7.3)	2.10 (0.84–5.29)
G/A and A/A	294 (34.5)	79 (36.9)	1.11 (0.82–1.52)	52 (39.4)	1.24 (0.85–1.80)	27 (32.9)	0.93 (0.58–1.51)
A frequency	0.189	0.213		0.220		0.201	

Abbreviation: HNPCC=hereditary non-polyposis colorectal cancer.
